# In-vitro examination of the positive inotropic effect of caffeine and taurine, the two most frequent active ingredients of energy drinks

**DOI:** 10.1186/s12872-017-0625-z

**Published:** 2017-08-10

**Authors:** R. Chaban, A. Kornberger, N. Branski, K. Buschmann, N. Stumpf, A. Beiras-Fernandez, C.F. Vahl

**Affiliations:** Department of Cardiothoracic and Vascular Surgery, University Hospital of Johannes Gutenberg University Mainz, Langenbeckstr. 1, 55131 Mainz, Germany

**Keywords:** Energy drinks, Taurine, Caffeine, Positive inotropic effect, Contractile force, Duration of contraction

## Abstract

**Background:**

Our study aimed to evaluate changes in the contractile behavior of human myocardium after exposure to caffeine and taurine, the main active ingredients of energy drinks (EDs), and to evaluate whether taurine exhibits any inotropic effect at all in the dosages commonly used in EDs.

**Methods:**

Myocardial tissue was removed from the right atrial appendages of patients undergoing cardiac surgery and prepared to obtain specimens measuring 4 mm in length. A total of 92 specimens were exposed to electrical impulses at a frequency of 75 bpm for at least 40 min to elicit their maximum contractile force before measuring the isometric contractile force (ICF) and duration of contraction (CD). Following this, each specimen was treated with either taurine (group 1, *n* = 29), or caffeine (group 2, *n* = 31) or both (group 3, *n* = 32). After exposure, ICF and CD measuring were repeated. Post-treatment values were compared with pre-treatments values and indicated as percentages.

**Results:**

Exposure to taurine did not alter the contraction behavior of the specimens. Exposure to caffeine, in contrast, led to a significant increase in ICF (118 ± 03%, *p* < 0.01) und a marginal decrease in CD (95 ± 1.6%, *p* < 0.01). Exposure to a combination of caffeine and taurine also induced a statistically significant increase in ICF (124 ± 4%, *p* < 0.01) and a subtle reduction in CD (92 ± 1.4%, *p* < 0.01). The increase in ICF achieved by administration of caffeine was similar to that achieved by a combination of both caffeine and taurine (*p* = 0.2).

The relative ICF levels achieved by administration of caffeine and a combination of taurine and caffeine, respectively, were both significantly higher (*p* < 0.01) than the ICF resulting from exposure to taurine only.

**Conclusion:**

While caffeine altered the contraction behavior of the specimen significantly in our in-vitro model, taurine did not exhibit a significant effect. Adding taurine to caffeine did not significantly enhance or reduce the effect of caffeine.

## Background

Energy drinks (EDs) have gained enormous popularity and are claimed to improve mental as well as physical performance [1–3]. Whether they exert a relevant positive inotropic effect or not, has not been conclusively established yet, even though an ample body of literature has been compiled on a variety of different cardiovascular effects attributed to EDs.

Typically, 100 ml of EDs contain about 32 mg caffeine, 400 mg taurine, 11 g glucose and vitamin B complex. A variety of other ingredients such as vitamins, minerals or herbal additives may also be found. Of these ingredients, caffeine and taurine are believed to exert a direct effect on the myocardium. According to a recent study, consumers of EDs are estimated to add up to 150–200 mg of caffeine per day to their diet from these products alone [4].

Used by humans for centuries, caffeine (1,3,7-trimethylpurine-2,6-dione) is still considered the number one stimulant in the world [5]. After oral ingestion, caffeine is rapidly absorbed and reaches its maximum blood and cerebral concentrations after 30–60 min, due to its water/fat solubility. Caffeine clearness varies widely between and within individuals due to its dependency on a specific form of cytochrome P450 oxidase and to a variety of interactions with nutrients und medications [6]. The half-life of caffeine is 2–8 h, and physiological blood-concentrations were reported to be up to 12 μg/ml [7].

Taurine (2-aminoethane sulfonic acid) is a ubiquitous amino acid. It is found in particularly high concentrations in cardiac and skeletal muscle [8, 9]. Taurine has also been found to be associated with positive inotropic and anti-arrhythmic effects through its Involvement in calcium hemostasis and was even suggested as a treatment for heart failure [10–14].

In-vivo investigation of the hemodynamic responses to EDs yielded inconsistent results. The studies performed to date examined different types of EDs, applying different study designs and evaluating different outcome parameters. Grasser et al. [15], for example, reported an elevation in blood pressure, heart rate and cardiac output after ingestion of one can of *Red Bull®* in comparison with the same amount of water and suggested an overall negative hemodynamic profile of the EDs due to a combination of an augmented work load to the heart and a reduced cerebral blood flow velocity due to elevated cerebrovascular resistance. Svatikova et al. [16] reported significant increases in systolic and diastolic blood pressure as well as in norepinephrine levels but no significant difference in heart rate increases between a group ingesting EDs and controls ingesting placebo. Pincomb et al. found that exposure to caffeine at a dose of 3.3 mg/kg for two days resulted in increases of blood pressure and vascular resistance as well as a decrease in heart rate [17], but failed to demonstrate a relevant increase in cardiac output or contractility. These findings are in keeping with those of other studies [18].

A positive inotropic effect of EDs was, in particular, demonstrated by imaging studies. An echocardiographic study published by Menci et al. [19] demonstrated an increase in left and right ventricular function one hour after intake of EDs containing taurine and caffeine, which led the authors to suggest a positive inotropic effect exerted by the substances contained in EDs. Baum et al. [20], also using echocardiography, demonstrated that fractional shortening and stroke volume increased significantly after consumption of *Red Bull®* containing caffeine and taurine in comparison with controls receiving variants of the same EDs containing caffeine only or neither caffeine nor taurine, respectively. Doerner et al. [21], in contrast, applied cardiac magnetic resonance imaging (MRI) and cardiac MR-based strain analysis to determine the short-term effects of EDs containing caffeine and taurine. In keeping with the echocardiographic studies, their investigation also yielded a subtle but significant increase of myocardial contractility one hour after consumption of EDs containing caffeine and taurine.

In order to supplement these in-vivo and imaging studies and to contribute to elucidating the positive inotropic effects of EDs, we applied an in-vitro model to evaluate the changes in contractile behavior of human myocardium after exposure to caffeine and taurine. Our experimental set-up aided us in understanding the positive inotropic effect of EDs and allowed us to distinguish between the respective effects of taurine and caffeine.

## Methods

The study was conducted with permission of the Ethics Board of Rheinland Pfalz, Germany, and after obtaining individual written consent from the patients. We used tissue samples from the right atrial appendages, which were removed and discarded in the course of cannulation of the right atrium for cardiopulmonary bypass in patients undergoing cardiac surgery at our hospital. Patients with severe cardiomyopathy, inflammatory or infective cardiac disease or similar conditions, which deemed to exert a relevant influence on the contractile behavior of the myocardium, were excluded.

Immediately after obtaining the tissues in the operating room, they were transferred to our laboratory in cold (4 °C) histidine-tryptophan-ketoglutarate (HTK) solution (“Kardioplegische Lösung”, The University Medical Center of the Johannes Gutenberg University, Mainz, Germany). There, they were manually prepared under the microscope without delay to yield muscle specimens measuring 4 × 0.5 × 0.5 mm. These muscle specimens were stored for 2–48 h in cold (4 °C) HTK solution and washed with Krebs–Henseleit solution (KH solution) before testing.

For the test, each muscle specimen was mounted horizontally between the tweezers of a muscle investigation apparatus (“Standard System for Muscle Investigation”, SH Heidelberg, 69,118 Heidelberg, Germany) and exposed to a continuous flow of warm (32°) KH solution steamed with a mix of 95% O2 and 5% Co2. Following accurate baseline length measuring (slack length), each specimen was stretched to 110% of its slack length. Then electrical impulses were applied at a frequency of 75 bpm. The voltage was gradually increased until the maximum isometric force of the specimen was reached. Afterwards, the specimens were left to rest for 30 to 60 min to allow them to reach a steady state before we proceeded to record our trails. The analog signal from the muscle investigation apparatus was transmitted to a digital-analog-converter (PicoScope 2204A, Pico Technology, Cambridgeshire, UK) by means of a BNC cable and recorded with the PicoLog Software (Pico Technology, Cambridgeshire, UK).

At the beginning of each trail, a period of 10 min was recorded, during which the specimens were exposed to continuous stimulation as indicated above. Specimens that exhibited a weak or unstable contractile force during the 10-min control period were excluded.

Following this, the specimens were subjected to one of three treatments using concentrations of taurine and caffeine that were about twice as high as the maximum plasma levels reported in humans after physiological exposure to taurine and caffeine. Group 1 was exposed to taurine (230 μg/ml), and group 2 to caffeine (20 μg/ml). Group 3 was exposed to a combination of taurine (230 μg/ml) and caffeine (20 μg/ml).

**Fig. 1:**
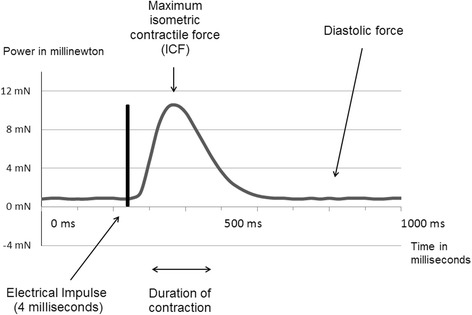
Recording of the forces occurring during one contraction, following electrical stimulation of a myocardial sample. The maximum isometric contractile force, diastolic force and the duration of contraction were recorded

**Fig. 2:**
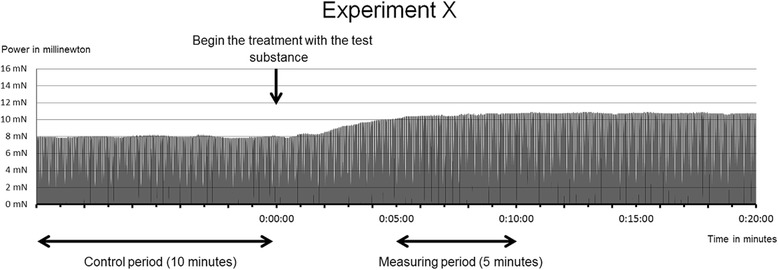
This figure shows the composition of each trail. The samples were exposed to electrical stimulation for at least 40 min before recording was started and continued for 10 min (control period). Then the test substance was applied, and after 5 min, measuring was recommenced and continued for 5 min (measuring period)

5 min after application of taurine and/or caffeine, the ICF and the CD developed by the specimen were registered for a period of 5 min (measuring period) (see Figs. 1 and 2).

### Statistical analysis

Analysis and data collection were performed using Excel 2010 software (Microsoft, Redmond, Washington, United States). For each trail, the mean and standard deviation of the average isometric contractile force during the control period (ICFc) and during the measuring period (ICFm) were determined. The relative ICF was then calculated according to the following equation:

$$ \mathrm{Relative}\ ICF\left(\%\right)=100\times \mathrm{ICFm}/\mathrm{ICFc} $$

The relative duration of contraction (CD) was calculated similarly.

Specimens that exhibited a weak or unstable contractile force during the 10-min control period were excluded. For this purpose, a threshold of 0.15mN was set for the ICFc and a threshold of 5% was set for its standard deviation.

Mean relative ICFs and the respective SEM were calculated for the three groups and compared using “R” Version 3.3.3 (The R Foundation for Statistical Computing) to conduct Wilcoxon rank sum tests and calculate *p*-values. A threshold of 0.01 was chosen for significance.

## Results

A total of 92 successful experiments were conducted in specimens obtained from 45 patients. 29, 31 and 32 trails were recorded for group 1 (taurine only), group 2 (caffeine only) and group 3 (taurine + caffeine), respectively.

The average patient age was 69.8 ± 9 years. The medical profiles and medications of the patients (*n* = 45) are summarized in Table 1.

**Table 1:** Summary of the medical profiles and medications of the patients

Aortic valve replacement	12	(27%)
Mitral valve repair/replacement	3	(7%)
Coronary artery bypass	33	(73%)
Coronary artery disease	37	(82%)
Atrial fibrillation	5	(11%)
Severly reduced EF	0	(0%)
Moderatly and slightly reduced EF	11	(24%)
Diabetes mellitus	19	(42%)
Severe aortic stenosis	12	(27%)
Mitral valve insufficiency	3	(7%)
Arterial hypertension	42	(93%)
Renal insufficiency	3	(7%)
Aspirin	31	(69%)
Clopidogrel	10	(22%)
Beta blockers	23	(51%)
Statins	28	(62%)
Diuretics	16	(36%)
Metformin	7	(16%)
Thyroxine replacement therapy	2	(4%)
Antihypertensive medications	22	(49%)

The voltage required to provoke maximum ICF was 13.2 ± 4.6 V (mean ± SD) (range 10 – 20 V). The voltage required to provoke maximum ICF did not differ significantly between group 1 (13.2 ± 4.6 V), group 2 (13.8 ± 4.9 V) and group 3 (12.5 ± 4.3 V). The ICF measured during the control period was 8.1 ± 1.6mN (mean ± SEM), 11.3 ± 1.5mN and 11.6 ± 1.2mN in groups 1, 2 and 3, respectively. A significant difference in ICF during the control period was not present between the groups.

### Effect on isometric contractile force (ICF)

**Fig. 3:**
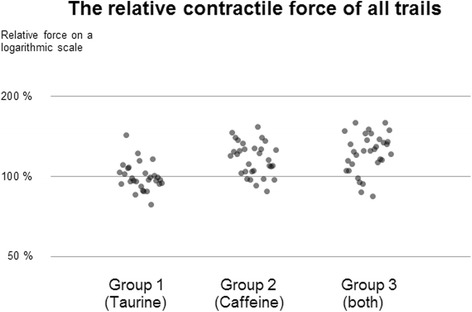
Relative contractile force values of all trails on a logarithmic scale. Group 2 and 3 showed significant increases (118 ± 03% and 124 ± 4% with *p*-value <0.01 for both), while group 1 showed no change at all

Exposure to taurine did not alter the ICF in group 1, where the ICF during the measuring period was 100 ± 02% (mean ± SEM) of the ICF during the control period. In group 2, exposure to caffeine led to a significant increase (*p* < 0.01) of the ICF to 118 ± 03%. In group 3, exposure to a combination of taurine and caffeine also led to a significant (*p* < 0.01) increase in ICF, which reached 124 ± 4% (see Fig. 3).

The relative ICF levels achieved by administration of caffeine and a combination of taurine and caffeine, respectively, were both significantly higher (*p* < 0.01) than the ICF resulting from exposure to taurine only. There was no significant difference in relative ICF between administration of caffeine and a combination of caffeine and taurine (groups 2 and 3).

### Effect on the duration of contraction (CD)

**Fig. 4:**
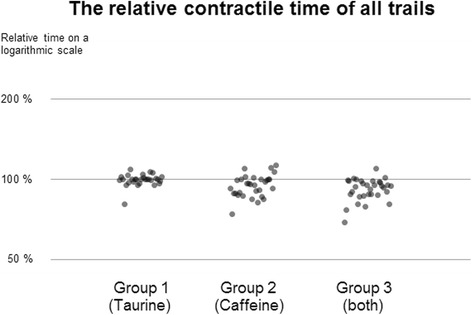
Relative durations of contraction (CD) of all trails on a logarithmic scale. Group 2 and 3 showed significant albeit small decreases (95 ± 1.6% and 92 ± 1.4% with *p*-value <0.01 for both) while group 1 showed no change at all

Treatment with caffeine caused a statistically significant, albeit small reduction of the duration of contraction (CD) to 95 ± 1.6% (*p* < 0.01). This also applies to the reduction seen in group 3 (92 ± 1.4%, *p* < 0.01). There was no significant difference between these two groups. In group 1, no significant change in the duration of contraction was registered (see Fig. 4).

## Discussion

Our experiment was performed in order to determine how the human myocardium responds to caffeine and taurine. The concentrations of caffeine and taurine were specifically chosen for an in-vitro, rather than an in-vivo setting and correspond to high but non-toxic plasma concentration levels of either substance. The concentration of caffeine (20 μg/ml) equals the plasma level after ingestion of 500 mg (approximately 4–5 cups of coffee) [22] and is well below the limit of toxicity established for caffeine (80–100 µg/ml) [23]. Mohammadreza et al. [24], studying the pharmacokinetics of oral taurine in healthy volunteers who ingested 4 mg of taurine, found a maximum plasma concentration of taurine of 86.1 ± 19.0 µg/ml after 1.5 ± 0.6 h from administration, with one participant reaching a plasma concentration of 112.6 µg/ml. The concentration we have chosen for our in-vitro model is approximately twice as high (230 µg/ml).

While EDs are usually marketed to a younger consumer group, the tissue samples that were available to us were from older individuals with cardiac disease requiring cardiac surgery. However, we excluded patients with a number of conditions that severely impact myocardial contractility and used only specimens from hearts with adequate in-vivo contractile properties. To avoid bias caused by samples severely damaged in the course of harvesting or transport, we also discarded tissue specimens that showed inadequate contractile behavior during the control period.

Moreover, our model was designed to determine whether or not a difference in contractile force is brought about by exposure to caffeine and/or taurine rather than to measure absolute contractile force values. Therefore, the use of myocardium from patients that differ from the typical target group of EDs was considered acceptable though it may be assumed that the response of myocytes from healthy young individuals would even be more pronounced than the response we elicited in our tissue specimens. For the same reasons, the use of atrial rather than ventricular tissue was considered acceptable.

Taking into account the results of an ample body of older literature on the mechanisms by which caffeine affects the cardiovascular system, and sharing the view expressed by Doerner et al. [25], we expected the inotropic effect of EDs, if any, to be caused by taurine rather than by caffeine. Leite-Moreira et al. [26] for example, examined the effect of caffeine on left ventricular contraction and relaxation in a dog model and found that intravenous administration of 50 mg/kg caffeine had no inotropic effect. Fujii et al. [27] administered caffeine into the coronary arteries of failing rat hearts at a dose yielding approximately the maximum blood concentration after consumption of a cup of coffee or tea and found that caffeine depressed left ventricular systolic and diastolic functions. In-vitro studies of the impact of caffeine on different types of animal myocardium also date back several decades and yielded conflicting results. While some reported a temporary positive inotropic response followed by a sustained negative effect [28–30], others suggested positive inotropic responses [31, 32]. From a number of in-vivo studies, no positive effect of caffeine on cardiac contractility was reported [17, 33].

Our results, with statistical significant differences in ICF between the control and the measuring period, seen only in groups that received caffeine, suggest that a positive inotropic effect of EDs must be attributed to caffeine rather than taurine. This is in keeping with recent findings according to which the cardiovascular responses to the ingestion of EDs are best explained by the actions of caffeine and sugar, with little influence from other ingredients, even though an effect from other active ingredients such as taurine cannot be excluded [34].

Doerner et al. [25], using MRI, recently found the left ventricular systolic volume (LVSV) and left ventricular end diastolic volume (LVEDV) significantly increased in their test subjects one hour after consumption of an ED containing caffeine and taurine, whereas the LVEDV was significantly decreased and the LVSV showed no significant change after consumption of EDs containing caffeine only. In both groups, the left ventricular ejection fraction (LVEF) showed no significant changes.

Baum et al. [20], using echocardiography, also aimed at distinguishing between the effects of caffeine and taurine. In contrast to Doerner et al. [25], who observed their test subjects at rest, Baum et al. [20] performed echocardiographic examinations at baseline, before exercise and in the regeneration period after exercise. After administration of EDs containing taurine and caffeine, the left ventricular end systolic diameter (LVESD) was found significantly decreased and the fractional shortening significantly increased, leading to a significant increment in stroke volume. In the control groups treated with placebo or EDs containing caffeine only, no significant alterations were registered, which also suggests that an increase in contractile force after consumption of EDs requires the presence of taurine. In our trial, the difference between the contractile force values obtained during the control period and the measuring period was not statistically significant in samples treated with taurine only. Adding taurine to caffeine did not yield a statistically significant alteration of the test result in comparison with administration of caffeine only, although a small increase in contractile force was observed.

In contrast to a number of other studies, we eliminated the impact of other ingredients found in EDs and concentrated only on caffeine and taurine, the two active components to which cardiovascular effects are generally attributed, which allowed us to reach accurate and separate results for each of these substances.

Our experimental model also allowed us to eliminate the impact of a variety of neurological, hormonal and other endogenous factors that cannot be eliminated in an in-vivo study. This is extremely valuable when it comes to a substance like caffeine, which, as a potent stimulant, strongly interferes with the sympathic/parasympatic regulation of vascular tone and cardiac function, which results in modified preload and afterload and affects the cardiac output.

Identifying effects on the myocardial tissue as such would be difficult in an in-vivo trial in humans. By comparing the ICF in the same specimen before and after application of caffeine and/or taurine, we avoided incoherence due to individual differences seen even in the same subject (different specimens from the same subject exhibit different ICF).

Our study provides no evidence whatsoever as far as the relevance of the observed positive inotropic effect in-vivo is concerned. Though the difference between the control period and the measuring period in the groups exposed to caffeine is statistically relevant, a relative increase to 128% in vitro resulting from a caffeine concentration that corresponds to twice the maximum plasma level reported in humans after physiological exposure may be neglectable in vivo.

Due to the nature of our in-vitro model, our results are intended to complement rather than contradict the results of the in-vivo work we used as a background for our discussion.

### Limitations

The muscle samples used in our trial were from elderly patients with cardiac conditions requiring surgery such as cardiovascular disease because myocardial tissue from healthy young individuals is not readily available. For the same reason, our specimens were from the cannulation site in the right atrium appendage rather than the ventricular myocardium. However, as we aimed at demonstrating only the presence or absence of an inotropic effect rather than quantifying its magnitude, differences between older and younger, healthy and diseased, as well as ventricular and atrial myocardium were neglectable. Moreover, specimens with inappropriate contraction behavior were excluded.

## Conclusions

While caffeine altered the contraction behavior of the specimen significantly in our in-vitro model, taurine did not exhibit a significant effect. Adding taurine to caffeine did not significantly enhance or reduce the effect of caffeine. These results should contribute to a better public awareness regarding the growing consume of energy drinks and its effects on public health.

## Abbreviations

CD: Duration of contraction
EDs: Energy drinks
ICF: Isometric contractile force
