# Correction to: “in-vitro examination of the positive inotropic effect of caffeine and taurine, the two most frequent active ingredients of energy drinks”

**DOI:** 10.1186/s12872-019-1012-8

**Published:** 2019-02-04

**Authors:** R. Chaban, A. Kornberger, N. Branski, K. Buschmann, N. Stumpf, A. Beiras-Fernandez, C. F. Vahl

**Affiliations:** Department of Cardiothoracic and Vascular Surgery, University Hospital of Johannes Gutenberg University Mainz, Langenbeckstr. 1, 55131 Mainz, Germany

**Correction to: BMC Cardiovascular Disorders (2017) 17:220**

**https://doi.org/10.1186/s12872-017-0625-z**

Following publication of the original article [1], the authors of the above mentioned article would like to declare that this work includes results, which are part of the medical Thesis of the co-author: Ms. Nicola Branski.
